# Expression and Predictive Significance of FHL1 and SLIT3 in Surgically Resected Lung Adenocarcinoma

**DOI:** 10.2174/1386207326666230208124028

**Published:** 2023-06-15

**Authors:** Jinjing Song, Kai Liang, Tongtong Wei, Li Li, Zhiguang Huang, Gang Chen, Naiquan Mao, Jie Yang

**Affiliations:** 1 Department of Pharmacology, School of Pharmacy, Guangxi Medical University, Nanning 530021, Guangxi, People’s Republic of China;; 2 Department of Thoracic Tumor Surgery, The Affiliated Cancer Hospital of Guangxi Medical University, Nanning 530021, Guangxi, People’s Republic of China;; 3 Department of Pathology, The First Affiliated Hospital of Guangxi Medical University, Nanning 530021, Guangxi, People's Republic of China.

**Keywords:** Lung adenocarcinoma, RNA sequencing, differentially expressed genes, prognosis, surgery, clinical specimen

## Abstract

**Objective:**

Lung adenocarcinoma (LUAD) is the most common type of lung cancer. However, predictive biomarkers for early efficacy and prognosis evaluation in patients with surgically resected LUAD are not completely explained.

**Methods:**

Differentially expressed genes (DEGs), gene ontology (GO) and Kyoto Encyclopedia of Genes and Genomes (KEGG) were identified by RNA sequencing (RNA-Seq) between thirteen LUAD tissues and five normal lung tissues. The expression of DEGs was confirmed by qRT-PCR and a validated cohort from GEPIA. Protein-protein interaction (PPI) network of the top 5% DEGs was constructed by STRING and visualized in Cytoscape. Immunofluorescence results were acquired from clinical specimens from LUAD patients. The expression of FHL1 was analyzed by ImageJ. Survival analysis was performed using the GEPIA dataset.

**Results:**

Consistent with the RNA-Seq data, validation of DEGs expression by qRT-PCR and GEPIA cohort showed that FHL1 and SLIT3 were down-regulated in LUAD patient tissues compared with non-tumor tissues. Moreover, FHL1 was significantly reduced in LUAD cell lines compared to the bronchial epithelium cell line (*P <* 0.01). However, SLIT3 was elevated in A549 and H1299 cells (wide type EGFR) (*P <* 0.05) while decreased in HCC827 and PC9 cells (mutant EGFR) compared to BESA-2B cells (*P <* 0.01). PPI network revealed the most significant cluster with 10 nodes and 43 edges. Immunofluorescent staining also showed that the expression of FHL1 was lower in LUAD tissues compared with that in normal lung tissues (*P <* 0.01). The expressions of SLIT3 and FHL1 were positively correlated. Specifically, the higher expression level of SLIT3 and FHL1 independently predicted a better prognosis (*P <* 0.01 or *P <* 0.05).

**Conclusion:**

Our findings provide two novel candidates, FHL1 and SLIT3, for prognostic evaluation and treatments after surgery.

## 1 INTRODUCTION

Lung adenocarcinoma (LUAD) accounts for approximately 40% of lung cancer [1] with increasing morbidity and mortality during past decades [2]. Most patients with LUAD are diagnosed at late stages (stage III and IV) with local and distant metastatic dissemination [3], which restricts the surgical options, thus limiting the survival benefit for LUAD patients [4]. Furthermore, even if patients with early-stage LUAD have undergone radical resection, the prognosis is still poor due to rapid relapse. About 50% of patients have tumor recurrence or metastasis within 5 years after surgery, and most postoperative-related recurrences or metastases occur within 1 year [5]. Therefore, it is an urgent requirement for predictive biomarkers for early efficacy and prognosis evaluation in patients with surgically resected LUAD, which will be useful to select the adjuvant therapy in time and improve the outcome of patients.

In the past decade, an increasing number of studies have identified many genes as prognostic biomarker candidates for lung cancer, including in LUAD. Scafoglio *et al.* found that SGLT2 (sodium-dependent glucose transporter 2) is highly expressed in lung premalignancy and early-stage/low-grade LUAD using a combination of immunohistochemistry and Me4FDG PET. Inhibiting SGLT2 by Gliflozins greatly reduced tumor growth and prolonged survival in mouse models, suggesting that SGLT2 is a potential diagnostic and therapeutic target for early-stage LUAD [6]. Moreover, Lissa *et al.* quantified HOXA9 (Homeobox A9) promoter methylation in formaldehyde fixed paraffin embedded (FFPE) samples of stage I lung adenocarcinoma patients by droplet digital PCR-based DNA methylation assay and found that high HOXA9 methylation alone or combined with blood vessel invasion (BVI) could potentially inform a higher risk of aggression or a worse outcome of patients with stage I LUAD [7]. Moreover, transcriptome profiling, such as RNA-seq and microarray, is generally utilized to discover new predictive biomarkers for the diagnosis, prognosis and efficacy assessment of lung cancer. Currently, RNA-seq is the preferred method to analyze gene expression patterns at the transcriptional level, since it obtains high accuracy, sensitivity and specificity, and broadens dynamic range [8, 9]. Furthermore, several genes (DLGAP5, KIF11, *etc*.) obtained by RNA-seq from the GEO dataset were demonstrated to be closely related to survival in LUAD and may serve as potential prognostic markers of LUAD [10]. Recently, single-cell RNA-seq (scRNA-seq) has provided a powerful tool for molecular biomarker discovery in tumors. Compared with RNA-seq technology, 10× scRNA-seq has relatively good performance in exploring tumor heterogeneity and distinguishing the molecular characteristics in each cell type [11, 12], but has some limitations, such as only sequencing the 3′ end, expensive cost, and relatively low coverage [11]. For example, based on the integrated analysis of RNA-seq and 10× scRNA-seq data of LUAD patients, Chen *et al.* identified that 51 genes were significantly changed in LUAD cells compared with alveolar cells and higher expression levels of HMGA1 and EMC6 were associated with poorer prognosis [13]. Besides, a sixteen-gene prognostic biomarker (LINC00908, PITX3, *etc*.) has been recently identified for LUAD by using a machine learning method [14]. However, there is still a lack of reliable and reproducible biomarkers to transform medical practice and care in patients with surgically resected LUAD.

In the present study, we collected surgically resected specimens from LUAD patients and utilized high throughput RNA-seq technology aiming to identify DEGs between LUAD tissues and normal lung tissues.

## 2 MATERIALS AND METHODS

### 2.1 Tissue Acquisition in Patients

Following Institutional Review Board (IRB) approval (No. 029, 2018) of Guangxi Medical University, 24 LUAD tissues and matched non-cancerous tissues were prospectively collected from patients undergoing surgical resection with signed informed consent from Dec 2018 to May 2019 in the Affiliated Tumor Hospital of Guangxi Medical University. Moreover, formaldehyde fixed paraffin embedded LUAD tissues and paired noncancerous tissues were collected from patients undergoing surgery from Jan 2013 to Dec 2018. Selection criteria included: (1) pathologically confirmed LUAD; (2) patients who did not receive either chemotherapy or radiotherapy before surgery; (3) patients without other tumor history; (4) patients with a complete medical record.

### 2.2 RNA Extraction, Quantification and Qualification

RNA extraction was performed using TRIzol reagent (Invitrogen, America) following the manual procedures. The concentration and purity were measured by NanoDrop 2000 (Thermo Fisher Scientific, Wilmington, DE). To determine RNA integrity, the RNA Nano 6000 Assay Kit of the Agilent Bioanalyzer 2100 system (Agilent Technologies, CA, USA) was conducted for evaluation.

### 2.3 Paired-end Library Preparation, Library Quality Control and Illumina Sequencing

To construct a useful cDNA library, eukaryotic mRNA was enriched with magnetic beads with Oligo (dT), then a fragmentation buffer was added to make mRNA fragmentation at random. The mRNA was used as a template to synthesize the first cDNA strand with random hexamers, then buffer, dNTPs, RNase H and DNA polymerase I were added to synthesize the second cDNA strand. AMPure XP beads were used to purify cDNA, and the purified double-stranded cDNA was end-repaired with the addition of A tail and connected to sequencing joints. Then AMPure XP beads were used to select the size of segments. Finally, a cDNA library was obtained through PCR enrichment and the quality of the library was tested. Qubit2.0 was used for preliminary quantification, and Agilent 2100 was used to detect the size of the library. The next experiment would be carried out only after the library size meets the expectation. The quantitative PCR (Q-PCR) was used to accurately quantify the effective concentration of library (> 2 nM). High-throughput sequencing was performed using NovaSeq 6000 (lllumina, San Diego, USA).

### 2.4 Data Preprocessing and Identification of Differentially Expressed Genes Identification (DEGs)

RNA-seq reads were aligned to the human genome (GRCh38/hg38) using HISAT2 [15]. Mapped reads were assembled and quantified by StringTie [16]. Differential expression was determined *via* DESeq2 [17] based on count values. Quantification of gene expression levels was estimated by fragments per kilobase of transcript per million fragments mapped reads (FPKM). The formula is shown as follows:


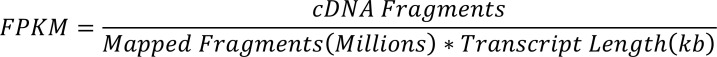


P-values were corrected by False Discovery Rate (FDR). The significant differential expression means fold change > 2 and FDR < 0.05. Statistically significant DEGs were evaluated by GO annotation and KEGG analysis using GOseq R packages [18] and KOBAS [19] software, respectively.

### 2.5 Reverse Transcriptase Quantitative Real-time PCR

Reverse transcription was performed according to PrimeScript^™^ RT Master Mix (Takara, Code No. RR036A). The relative expression was measured using SYBR^®^ Green reagents for use on the 7500 Real Time PCR instrument (Applied Biosystem, CA, USA). Primer sequences used in the present study are listed in Table **S1**.

### 2.6 Protein-protein Interaction Network (PPI Network) Construction of DEGs and Identification of Hub Genes

A total of 328 DEGs that ranked top 5% in FPKM or fold change (FC) were imported into the STRING database (https://string-db.org/) to construct a PPI network, in which disconnected nodes were hidden. The minimum required interaction score: medium confidence (0.400). The Cytoscape software was used to visualize the PPI network. Moreover, Molecular Complex Detection (MCODE) was applied to find the hub clusters of genes in the PPI network [20]. The score of MCODE >3 and the number of nodes >5 were set as cut-off criteria with the default parameters.

### 2.7 Immunofluorescent Staining

The histopathological sections were roasted at 70 °C for 5 h, dewaxed and hydrated by xylene, gradient ethanol and double steam water, and then treated with citric acid/sodium citrate buffer solution (pH = 6) under high pressure for antigen repair. After 5 min of high pressure, the sections were removed at room temperature. The repaired sections were washed twice with PBS and then sealed with 5% BSA at room temperature for 1 h. FHL1 primary antibody (Abcam, ab255828) was configured according to the instructions, and the slices were placed in a wet box and incubated at 4°C overnight. The next day, a wet box was placed at room temperature for 1 h, then the sections were washed with PBS 3 times, and the fluorescence secondary antibody Alexa555-Sheep anti-rat (CST, #4413) was incubated at 37°C for 1 h. After washed with PBS, sections were stained with DAPI, washed with PBS 3 times, and sealed with glycerol. FHL1 staining was observed and photographed in three random sights under a confocal laser scanning microscope, fluorescence intensity was analyzed by Image J and semi-quantitative analysis of mean fluorescence intensity was performed as previously reported [21, 22] (Table **S2**).

### 2.8 Survival Analysis

Overall survival(OS)plot of potential prognostic DEGs in LUAD was analyzed using the GEPIA database (Gene Expression Profiling Interactive Analysis, http://gepia.cancer-pku.cn), a web-based tool to deliver fast and customizable functionalities based on TCGA and GTEx data [23]. The parameters were set as follows: Group Cutoff: Median, Confidence Interval: 95%, log-rank p-value: <0.05.

### 2.9 Statistical Analysis

Differential expressed genes between LUAD tissues and matched normal lung tissues were analyzed by paired t-test and differential expressed genes among different cell lines were analyzed by ANOVA. Data are presented as the mean ± standard deviation (SD), and *P <* 0.05 (two‐sided) was considered the threshold for statistical significance. All analyses were performed using Prism6.0 or SPSS19.0 software.

## 3 RESULTS

### 3.1 Clinical Characteristics of Tissue Specimens

Five paired cancerous and non-cancerous tissues and eight individual tumor tissues were enrolled for further verification. Clinical characteristics of patients are summarized in Table **1**. There were five males (three of them had a history of smoking) and eight females with a mean age of 55.6 ± 5.2 years old. Seven patients harbored regional lymph node metastasis and no patients had distant metastasis. Only three patients had wild-type EGFR, among them, only one patient got both p53 and KRAS mutations, and the rest of the patients did not do the gene detection due to high cost.

### 3.2 GO and KEGG Analysis of DEGs

**Fig. (1):**
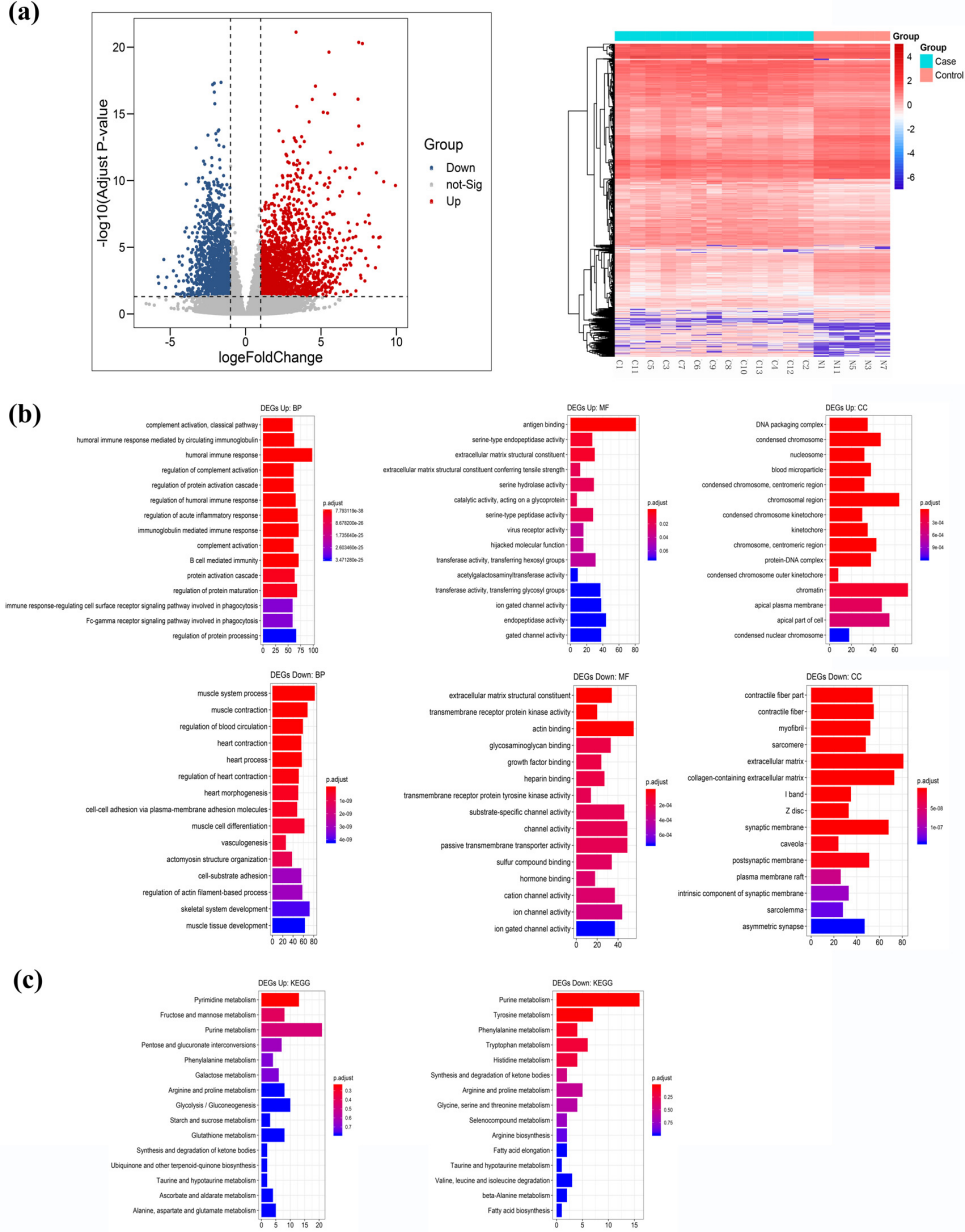
(**a**) Volcano Plot and Heatmap of DEGs. Red dots represent 1873 up-regulated genes and blue dots represent 1405 down-regulated genes. Case group refers to Cancerous tissues and Control group refers to non-cancerous tissues. (**b**) Gene Ontology (GO) analysis including BP (biological processes), MF (molecular function) and CC (cellular component) of up-regulated DEGs and down-regulated DEGs. (**c**) KEGG analysis of up-regulated DEGs and down-regulated DEGs.

A total of 3278 significant DEGs were identified, including 1873 up-regulated genes and 1405 down-regulated genes (Fig. **1a**). GO analysis showed that up-regulated DEGs were significantly enriched in humoral immune response for biological processes (BP); antigen binding for molecular function (MF); and chromatin for cellular component (CC), while down-regulated DEGs were significantly enriched in muscle system process, actin binding and extracellular matrix for BP, MF and CC respectively *(*Fig. **1b**). BP analysis indicated that IGHG1, IGHG4, FHL1 and SLIT3 participate in plentiful biological procedures. For instance, SLIT3 response to alcohol and negative regulation of growth; MFs of FHL1 were adherens junction, focal adhesion, cell-substrate junction and cell-substrate adherens junction.

KEGG pathway analysis showed the up-regulated DEGs were enriched in purine metabolism and pyrimidine metabolism, while down-regulated DEGs were significantly enriched in purine metabolism and tyrosine metabolism (Fig. **1c**). Among these, SLIT3 is a constituent part of Axon guidance, and FHL1 belongs to the JAK-STAT signaling pathway.

### 3.3 Construction of Protein-Protein Interaction Network (PPI Network)

**Fig. (2):**
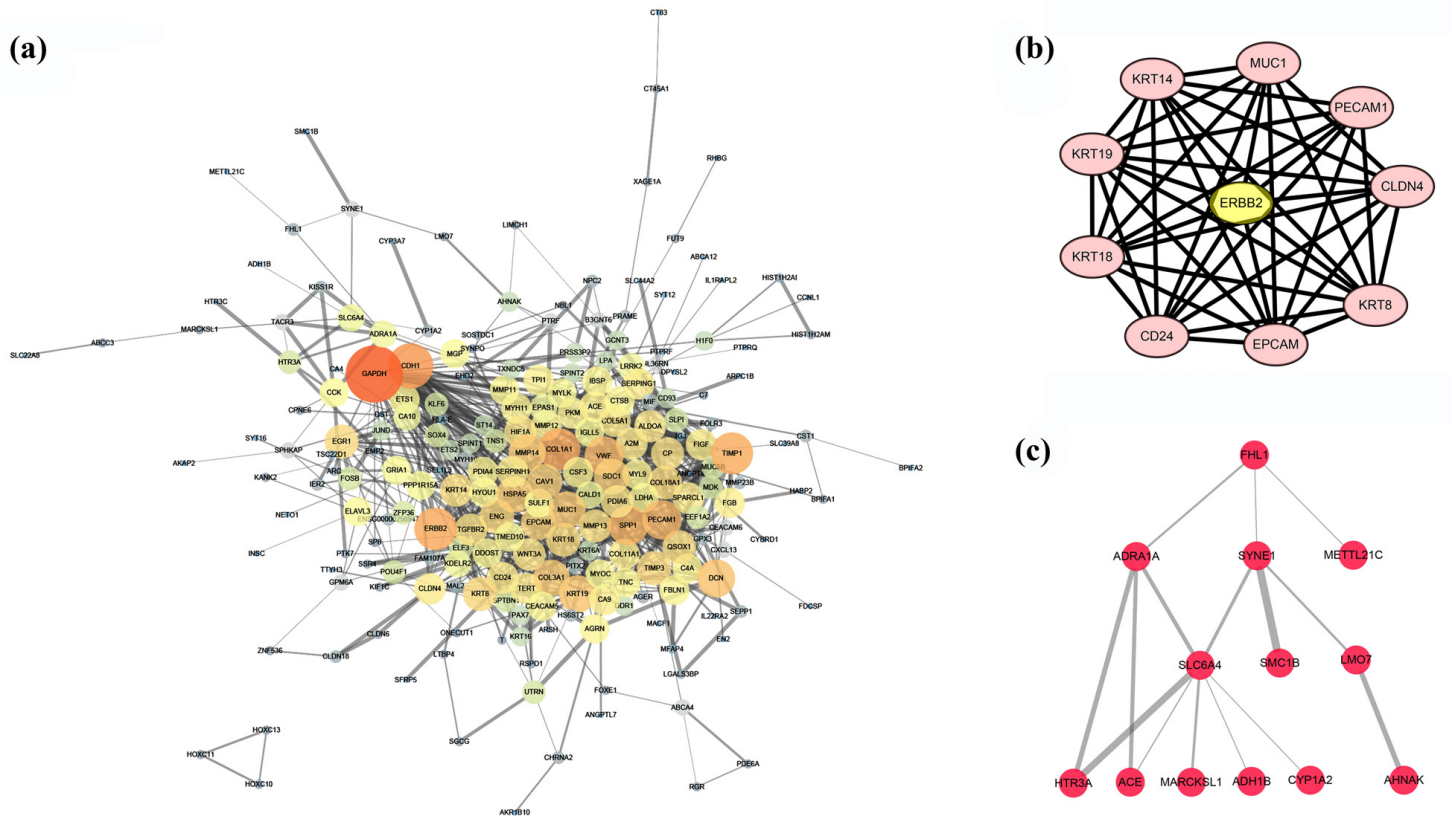
(**a**) The protein-protein interaction (PPI) network of 188 up-regulated and 140 down-regulated DEGs that ranked top 5% in FPKM or fold change (FC) with 221 nodes and 816 edges. (**b**) The most significant cluster generated by MCODE with 10 nodes and 43 edges. Small node size and dark node color represent low degree (number of edges connected to the node), while large edge size stands for a high combined score. (**c**) Proteins that interacted with FHL1 from the first to the third shell in the PPI network. The 1st shell interactors are the proteins directly interacted with FHL1, 2nd and 3rd shell interactors are the proteins interacted with FHL1 from the 1st shell within the PPI network.

A PPI network among DEGs with 221 nodes and 816 edges was constructed (Fig. **2a**). The most significant cluster (Fig. **2b**) had 10 nodes and 43 edges, though FHL1 was non-clustered in the results of the Molecular Complex Detection (MCODE) plugin from Cytoscape. In Fig. (**2c**), 1st shell proteins directly interacting with FHL1 were ADRA1A, SYNE1 and METTL21C. Second and 3rd shell proteins interacting with FHL1 from the 1st shell included SLC6A4, SMC1B, LMO7, *etc*.

### 3.4 Validation of DEGs Expression Profile by qRT-PCR in a Cohort Derived from GEPIA, Clinical Specimens, and LUAD Cell Lines

**Fig. (3):**
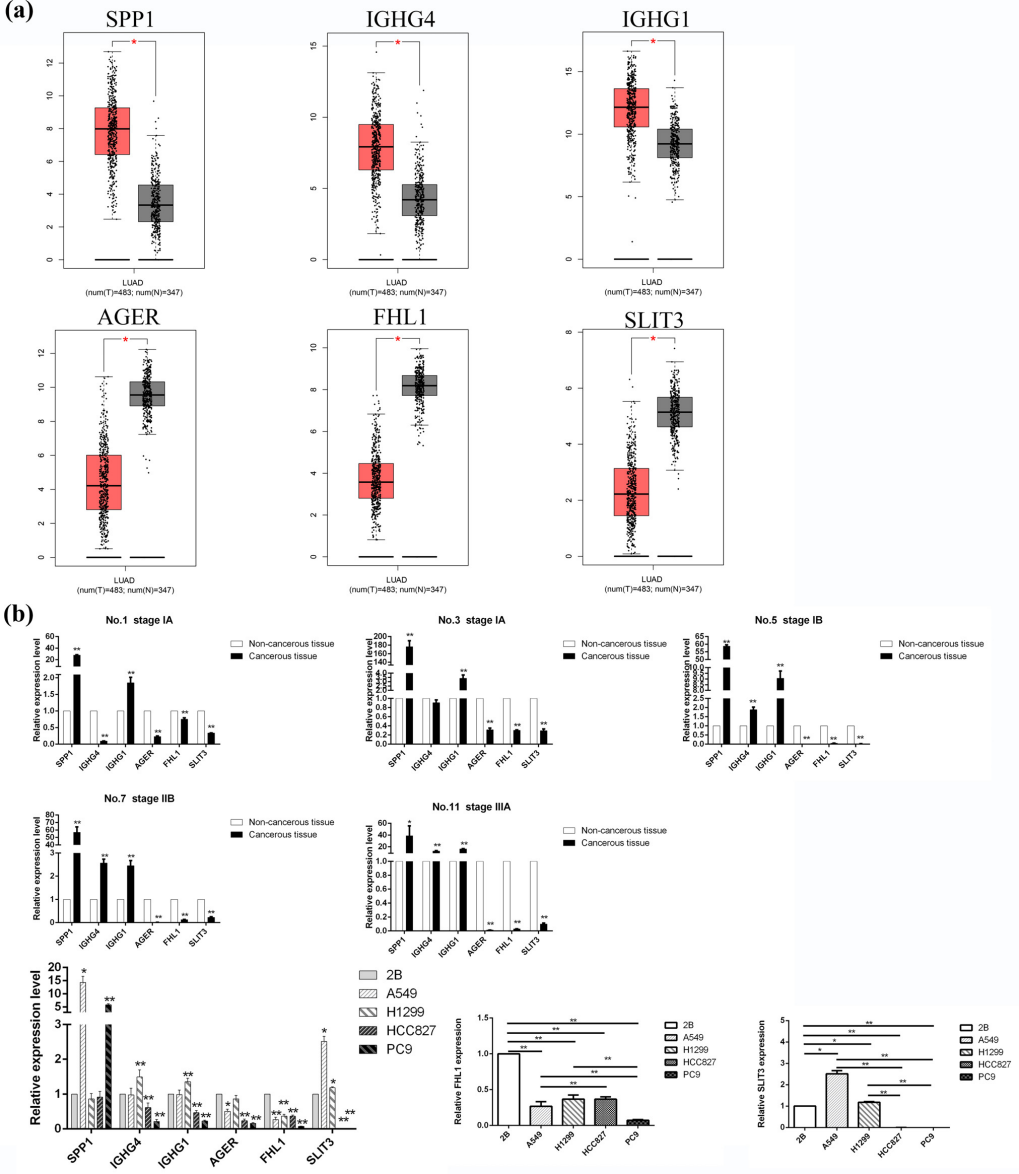
(**a**) Box plots from GEPIA demonstrate that the expression profiles of SPP1, IGHG4 and IGHG1 are up-regulated while AGER, FHL1 and SLIT3 are down-regulated in LUAD (*P < 0.05). (**b**) QRT-PCR results of SPP1, IGHG4, IGHG1, AGER, FHL1 and SLIT3 in five paired LUAD tissue samples compared with normal counterpart, and expression level in LUAD cell lines (A549, H1299, PC9, HCC827) compared with normal human bronchus epithelium cell line (BEAS‐2B), respectively (*P < 0.05, **P < 0.01).

Consistently, SPP1, IGHG4 and IGHG1 were up-regulated, while AGER, FHL1 and SLIT3 were down-regulated in LUAD tissues (n = 483) compared to normal lung tissues (n = 347) (Fig. **3a**). The expressions of SPP1, IGHG4, IGHG1, AGER, FHL1 and SLIT3 in 5 paired LUAD and normal lung tissues were in agreement with the RNA-Seq results (Fig. **3b**), except that IGHG4 was inversely down-regulated in sample No. 1 (stage IA) and showed no statistical significance in sample No. 3 (stage IA). FHL1 was significantly down-regulated in four LUAD cell lines (A549, H1299, HCC827 and PC9) compared with the normal lung epithelial cells BEAS‐2B (*P <* 0.01). Particularly, the most significant decline of FHL1 was seen inPC9 cells harboring an EGFR-activating mutation (exon 19 deletion). Moreover, SLIT3 was up-regulated in A549 and H1299 cells carrying wide-type EGFR (*P <* 0.05) while down-regulated in HCC827 and PC9 cells harboring EGFR-activating mutations (exon 19 deletion) (*P <* 0.01). Compared with BEAS‐2B cells, IGHG4 and IGHG1 were up-regulated in H1299 cells (*P <* 0.01) and down-regulated in HCC827 and PC9 cells (*P <* 0.01), SPP1 was up-regulated in A549 and PC9 cells (*P <* 0.05 or *P <* 0.01) and AGER was down-regulated in A549, HCC827 and PC9 cells (*P <* 0.05 or *P <* 0.01).

### 3.5 Differential Expression of FHL1 in Cancerous and Paired Noncancerous Tissues from LUAD Patients

As shown in Fig. (**4**), immunofluorescence staining results showed that FHL1 was mainly expressed in the cytoplasm in normal lung tissues while distributed in the cytoplasm and nucleus in LUAD tissues. Semi-quantitative analysis showed that the immunofluorescence intensity of FHL1 in LUAD tissues was significantly weaker than that in paired noncancerous tissues (*P <* 0.01), as well as the expression level of FHL1 in LUAD tissues was lower than that in normal lung tissues (*P <* 0.01).

### 3.6 Prognostic Significance of IGHG4, IGHG1, SPP1, AGER, FHL1 and SLIT3 in Surgically Resected LUAD

**Fig. (5):**
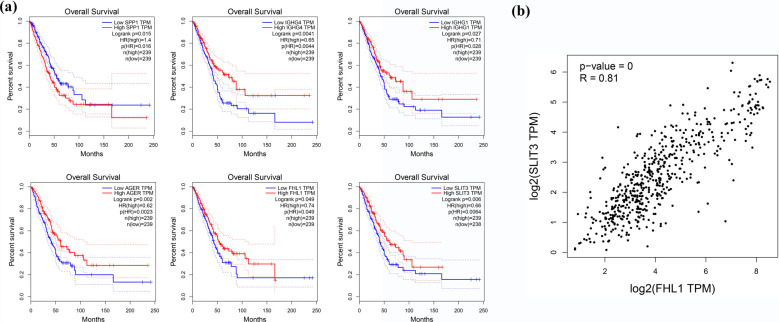
(**a**) Overall survival plots of SPP1, IGHG4, IGHG1, AGER, FHL1 and SLIT3 (*P* < 0.05) in LUAD. (**b**) FHL1 expression is correlated with SLIT3 expression in LUAD with Pearson correlation coefficient R=0.81 (*P* < 0.01).

The up-regulated and down-regulated DEGs that ranked top 10% both in log2 fold change and FPKM are listed in Table **2** and Table **3**. As listed, SPP1, IGHG4 and IGHG1 were up-regulated, while AGER, FHL1 and SLIT3 were down-regulated. Importantly, the high expression of IGHG4, IGHG1, AGER, FHL1 and SLIT3 was closely associated with increased OS (*P <* 0.05), whereas the high expression of SPP1 was significantly correlated to diminish OS (*P <* 0.05) *(*Fig. **5a**). Besides, FHL1 was correlated with SLIT3 in LUAD with Pearson correlation coefficient R=0.81 (*P <* 0.01) *(*Fig. **5b**).

## 4 DISCUSSION

In the present study, we identified two novel prognostic candidates, FHL1 and SLIT3, for surgically resected LUAD patients by using RNA-seq analysis in LUAD specimens. We also further validated these results in public datasets, clinical samples and cell lines. More importantly, low expression of FHL1 or SLIT3 may predict a poor outcome for LUAD patients after surgical treatment. Additionally, FHL1 and SLIT3 may participate in response to EGFR-TKI (Epidermal Growth Factor Receptor-Tyrosine Kinase inhibitor) in LUAD patients, although more efforts should be taken to explore this function and the underlying mechanisms.

Among 3278 DEGs, SPP1 and AGER ranked first in up-regulated and down-regulated DEGs, respectively, which signified worse outcomes for LUAD patients. A high expression of SPP1 and a low expression of AGER were associated with unfavorable OS in LUAD patients (*P <* 0.05), which was in accordance with a previous reported study based on the TCGA database [24]. Therefore, our findings reinforce that SPP1 and AGER may serve as potential biomarkers to predict the survival outcome of LUAD patients.

IGHG1 (immunoglobulin gamma-4 heavy chain) and IGHG4 (immunoglobulin γ-1 heavy chain) were two of the four human immunoglobulin G (IgG) subclass heavy chain (IGHG) genes. Traditionally, Immunoglobulins (*e.g*., IgG, IgM) were thought to be produced only by differentiated B lymphocytes, but our study has demonstrated that they might also express in non-hematopoietic human cancer cells, including epithelial lung cancers [25]. IGHG was also detected in the cytoplasm of lung squamous carcinoma tissue and adenocarcinoma cell line A549 [26]. Recently, IGHG1 has been reported to facilitate prostate cancer growth *via* the MEK/ERK/c-Myc pathway, while suppression of IGHG1 expression by siRNA led to growth inhibition and apoptosis induction [27, 28]. Conversely, high expression of IGHG1 was correlated with recurrence and functioned as a tumor suppressor in triple-negative breast cancer [29]. However, the role of IGHG1 in LUAD patients remains largely unknown. Furthermore, although IGHG4 (14q32.33) was identified as one of the most significantly down-regulated genes in three Taiwanese and one Caucasian ESCC cell lines compared with three normal tissues of esophagus, the function of IGHG4 in human carcinoma is still unclear. Interestingly, we found that IGHG4 expression was correlated with IGHG1 in LUAD.

The human FHL1 gene, located on chromosome Xq26, has been reported as a tumor suppressor [30]. FHL1 can be silenced by miR-410 or EZH2 epigenetically and regulates cancer cell growth [30-34], and is associated with transcriptional machinery [35]. The inhibitory effect of FHL1 on lung cancer cell growth has been reported *in vivo* and *in vitro* [36]. Contradictorily, several studies have shown that FHL1 might hinder the treatment. For example, the knockdown of FHL1 significantly enhanced the sensitivity of paclitaxel in hepatic carcinoma cells [37]. Moreover, the knockdown of FHL1 enhanced tumorigenesis *in vivo* and *in vitro*, which suggests that this protein could be a therapeutic target for treating head and neck squamous cell carcinoma [38]. FHL1-targeted intervention enhanced the sensitivity of AML cells to cytarabine, wherein its high expression powerfully predicted poor clinical outcomes in AML [39]. Recently, FHL1 was found to be phosphorylated by the cytosolic tyrosine kinase Src, which switched FHL1 from a tumor suppressor to a cell growth accelerator [35].

In our study, we illustrated that postoperative LUAD patients with high expression of FHL1 were associated with improved OS. Similarly, down-regulation of FHL1 was associated with a poor prognosis of esophageal squamous cell carcinoma and oral cancer [40, 41]. We also showed that FHL1 was significantly down-regulated in four LUAD cell lines (A549, H1299, HCC827 and PC9) compared with normal lung cell line BEAS‐2B. Besides, the protein level of FHL1 was lower than that in normal lung tissues and high expression of FHL1 was closely associated with increased OS. KEGG pathway analysis indicated that FHL1 was involved in the Janus kinase/signal transducer and activator of the transcription (JAK/STAT) signaling pathway, which was reported to be activated in NSCLC with EGFR mutation [42]. Therefore, our results suggest that a high level of FHL1 may serve as an early predictor of favorable outcomes in postoperative LUAD patients.

Human Slit Homolog 3 (SLIT3) is a secreted protein encoded by the SLIT3 gene. The tumor suppressive role of SLIT3 has been shown to inhibit cancer cell growth and invasion and migration in several tumors [43-46]. Additionally, SLIT3 silencing could lead to the enhanced invasive ability of A549 cells [46]. However, a comprehensive analysis addressing the clinicopathological and predictive significance of SLIT3 in cancers, particularly in LUAD, is still lacking. In this study, we demonstrated that SLIT3 was elevated in A549 and H1299 cells carrying wide-type EGFR while decreased in HCC827 and PC9 cells harboring EGFR-activating mutations compared with normal BEAS‐2B cells. Importantly, low expression of SLIT3 was significantly correlated to poor OS. Besides, a significant positive correlation between SLIT3 and FHL1 expression existed in LUAD. Therefore, our results suggest that higher expression SLIT3 is a novel prognostic indicator for better survival outcomes in postoperative LUAD patients. Whether SLIT3 is involved in the regulation of EGFR-TKI therapy in these patients is required further research.

Importantly, solid tumors are not simply clones of cancer cells. Instead, they are abnormal organs composed of multiple cell types and extracellular matrix [47]. Actually, SPP1 has been reported as a potential prognostic and immunotherapy biomarker, and correlated with tumor-infiltrating immune cells in multiple cancers [48-50]. High expression of IGHG1 also indicates more immune cell infiltration in glioma [51] and might be responsible for immune evasion in pancreatic carcinoma [52]. The expression of marker gene IGHG4 was found to be significantly higher in the diffuse subcluster developed from activated B cells which highly correlated with metastasis of colorectal cancer [53]. In addition, SLIT3 was identified as an active ligand secreted from CD36^+^ FBs that induced growth suppression of MDA-MB-231 breast cancer cells [54]. Based on our observations of upregulations of SPP1 and IGHG1/4 as well as downregulation of SLIT3, these reports suggest that the infiltration of immune cells may increase in surgically resected LUAD. However, is the infiltration of immune cells associated with the prognosis of patients with surgically resected LUAD? Some future work is needed to elucidate it.

## CONCLUSION

Our findings reveal that FHL1 and SLIT3 are two novel predictive biomarkers for early efficacy and prognosis evaluation in patients with surgically resected LUAD. Additionally, the key genes and functional enrichments may provide a valuable clue for investigating the molecular mechanisms underlying LUAD after surgical treatment. However, our study has some limitations, such as the contradictory roles between the increased expressions of IGHG1 and IGHG4 in LUAD tissues and their correlation with improved OS in LUAD patients. The regulatory functions and mechanisms of FHL1 and SLIT3 in postoperative LUAD patients also require further investigation. Our study does provide new evidence that FHL1 and SLIT3 may be promising prognostic biomarkers for efficacy and prognosis evaluation in LUAD patients undergoing surgery.

## Figures and Tables

**Fig. (4):**
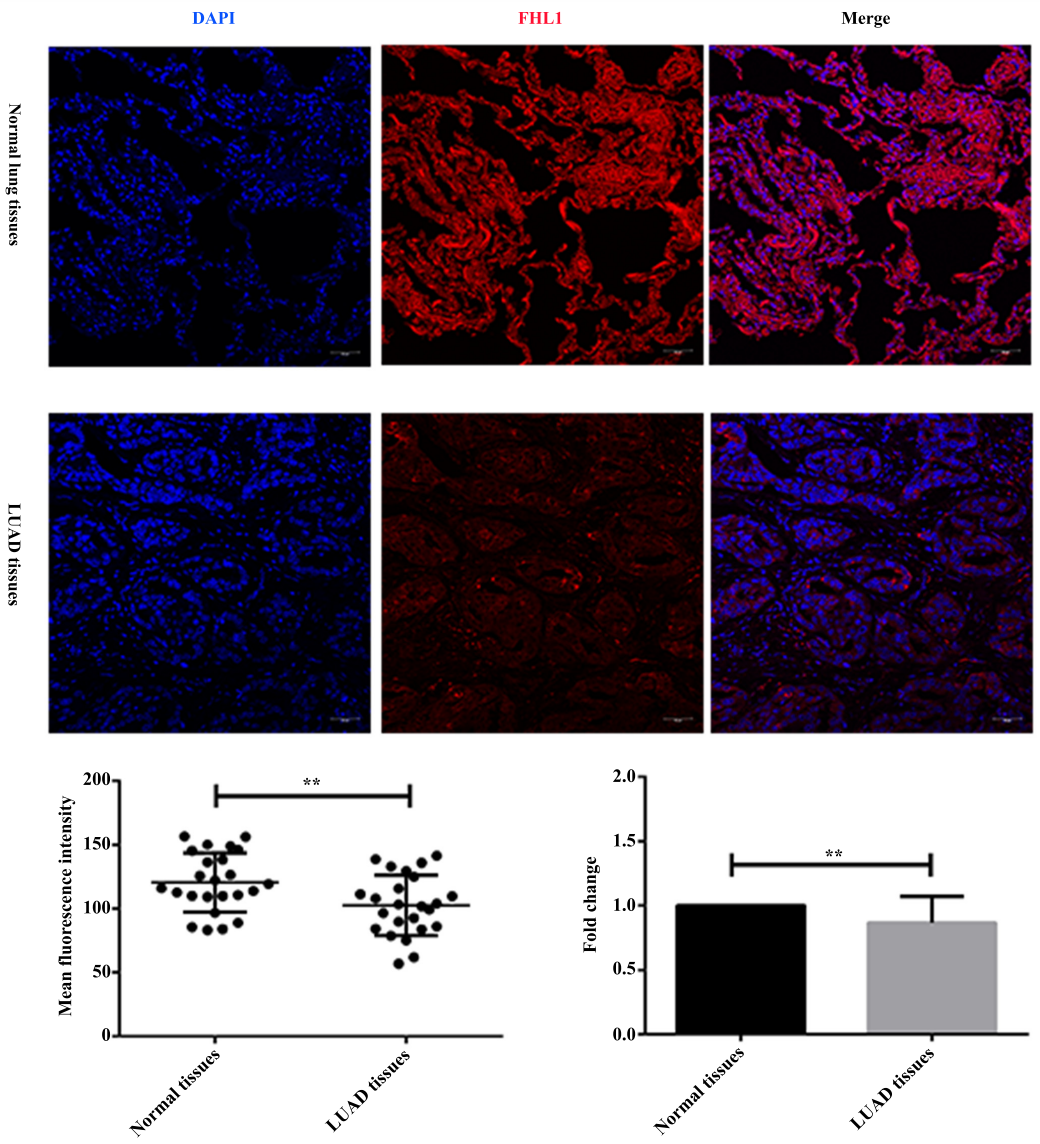
Immunofluorescence staining of FHL1 protein in paired normal lung tissues and LUAD tissues (×200) (*P < 0.05, **P < 0.01).

**Table 1:** Clinical characteristics of the patients.

Patient Number	Gender	Age	Tumor Size (cm^3^)	Histological Type	Clinical Stage	Stage	Smoking Status	p53	KRAS	EGFR
1	Female	57	2.5*2.0*2.0	Adenocarcinoma	PT1N0M0	IA	No	N/A	N/A	N/A
2	Male	64	1.5*1.5*1.5	Adenocarcinoma	PT2N0M0	IA	Yes	N/A	N/A	N/A
3	Female	47	2.5*1.5*1.5	Adenocarcinoma	PT1N0M0	IA	No	N/A	N/A	N/A
4	Male	60	1.0*0.5*0.7	Adenocarcinoma	PT2aN0M0	IB	No	N/A	N/A	N/A
5	Female	73	2.5*2.5*2.0	Adenocarcinoma	PT2N0M0	IB	No	N/A	N/A	Wild Type
6	Female	54	3.0*2.5*2.0	Adenocarcinoma	PT2N1M0	IIA	No	N/A	N/A	N/A
7	Female	58	3.5*2.5*2.0	Adenocarcinoma	PT2N0M0	IIB	No	N/A	N/A	N/A
8	Female	52	3.0*2.5*2.5	Adenocarcinoma	PT2N1M0	IIB	No	N/A	N/A	N/A
9	Female	59	6.5*6.0*3.5	Adenocarcinoma	PT3N1M0	IIIA	No	N/A	N/A	N/A
10	Female	55	2.0*2.0*1.8	Adenocarcinoma	PT2N2M0	IIIA	No	Mutant Type	Mutant Type	Wild Type
11	Male	59	6.5*6.0*3.5	Adenocarcinoma	PT3N1M0	IIIA	Yes	N/A	N/A	N/A
12	Male	52	7.5*7.0*5.0	Adenocarcinoma	PT4N2M0	IIIB	Yes	N/A	N/A	N/A
13	Male	53	3.5*3.0*2.5	Adenocarcinoma	PT3N2M0	IIIB	No	N/A	N/A	Wild Type

**Table 2:** Up-regulated DEGs that rank top 10% both in log_2_ fold change and FPKM.

**Gene**	**FPKM**	**Log_2_ Fold Change**	**Adjust *p*-value**
SPP1	11989.49	7.747563	5.34E-21
MMP11	3456.933	7.522945	9.19E-10
CEACAM5	8303.887	6.087853	1.17E-05
IGHG4	4847.922	5.747655	5.11E-07
CRABP2	2565.079	5.622687	7.47E-13
IGHV3-48	2199.743	5.379689	2.89E-08
IGHV1-3	3185.576	4.935735	7.80E-07
IGHV1-18	5542.457	4.916195	4.05E-07
IGLV3-10	2463.561	4.905078	1.74E-05
IGHG1	182981.3	4.483199	0.00022

**Table 3:** Down-regulated DEGs that rank top 10% both in log_2_ fold change and FPKM.

**Gene**	**FPKM**	**Log_2_ Fold Change**	**Adjust *p*-value**
AGER	8756.756	-3.68595	9.00E-05
RTKN2	2463.208	-3.58298	2.48E-05
CLDN18	3870.591	-3.42619	0.004997
FOSB	9765.474	-3.2627	0.044443
FMO2	3545.055	-3.18156	3.74E-08
ADH1B	6522.335	-3.13625	0.00012
CAVIN2	2904.494	-3.12773	1.20E-10
CAV1	9835.637	-3.01414	1.44E-05
FHL1	4327.047	-3.01156	1.22E-05
SLIT3	2333.855	-2.89089	7.14E-07

## Data Availability

The authors confirm that all data generated or analyzed during this study are included in this published article.

## LIST OF ABBREVIATIONS

LUAD: Lung Adenocarcinoma
DEGs: Differentially Expressed Genes
GO: Gene Ontology
KEGG: Kyoto Encyclopedia of Genes and Genomes
RNA-Seq: RNA Sequencing
PPI: Protein-protein Interaction
FFPE: Formaldehyde Fixed Paraffin Embedded
BVI: Blood Vessel Invasion
FC: Fold Change
OS: Overall Survival
