# Supplementary material for: Expression and Predictive Significance of FHL1 and SLIT3 in Surgically Resected Lung Adenocarcinoma
Source: Comb Chem High Throughput Screen. 2023 Jun 15;26(12):2226–37. doi: 10.2174/1386207326666230208124028 (PMC10339681; doi:10.2174/1386207326666230208124028)
Supplement: Supplementary file 1 [file CCHTS-26-2226_SD1.pdf]

Supplementary Material

Expression and Predictive Significance of FHL1 and SLIT3 in Surgically Resected Lung Adenocarcinoma

Jinjing Song[1#], Kai Liang[2#], Tongtong Wei[1], Li Li[1], Zhiguang Huang[3], Gang Chen[3], Naiquan Mao[2,*] and Jie Yang[1,*]

[1]*Department of Pharmacology, School of Pharmacy, Guangxi Medical University, Nanning 530021, Guangxi, People's Republic of China;* [2]*Department of Thoracic Tumor Surgery, The Affiliated Cancer Hospital of Guangxi Medical University, Nanning 530021, Guangxi, People's Republic of China;* [3]*Department of Pathology, The First Affiliated Hospital of Guangxi Medical University, Nanning 530021, Guangxi, People's Republic of China.*

Table 1. Primer sequence used in the present study.

Target	Forward primer sequence	Reverse primer sequence
**SPP1**	5′-TCACACATGGAAAGCGAGGAGTTG-3′	5′-ACTGTCCTTCCCACGGCTGTC-3′
**IGHG4**	5′-AGCTCAAGGCGGGACAGGTG-3′	5′-AGAGATGGAGGTGGATGCGTCAG-3′
**IGHG1**	5′-TGAGCCACGAAGACCCTGAGG-3′	5′-AGGACGGTGAGGACGCTGAC-3′
**AGER**	5′-CTCTCCTCAAATCCACTGGATG-3′	5′-CTATCTCAGGGAGGATCAGCA-3′
**FHL1**	5′-GACTGCTTCACCTGTAGTAACT-3′	5′-AGGTAACACACACAAAGCAATC-3′
**SLIT3**	5′-TCAATGCCAACAAGATCAACTG-3′	5′-CAAATGGGTTTTGGGCTAAGTG-3′

Table 2. Patient's information and IHC score from HPA database.

Patient ID	Gender	age	Classification	Antibody	Target protein	IHC Score
**2268**	Female	49	Normal	HPA001391	FHL1	Negative
**2417**	Male	59	Normal	HPA001391	FHL1	Low Positive
**2373**	Male	69	Normal	HPA001391	FHL1	Negative
**426**	Male	48	LUAD	HPA001391	FHL1	Negative
**461**	Male	60	LUAD	HPA001391	FHL1	Negative
**537**	Female	50	LUAD	HPA001391	FHL1	Negative
**1249**	Female	44	LUAD	HPA001391	FHL1	Negative
**1303**	Male	68	LUAD	HPA001391	FHL1	Negative
**1327**	Male	64	LUAD	HPA001391	FHL1	Negative
**1470**	Male	65	Normal	HPA051630	SLIT3	Low Positive
**1678**	Female	57	Normal	HPA051630	SLIT3	Low Positive
**2208**	Female	67	Normal	HPA051630	SLIT3	Negative
**1847**	Male	64	LUAD	HPA051630	SLIT3	Negative
**2393**	Female	54	LUAD	HPA051630	SLIT3	Negative
**2777**	Female	69	LUAD	HPA051630	SLIT3	Negative

**2888**	Male	67	LUAD	HPA051630	SLIT3	Negative
**3003**	Male	49	LUAD	HPA051630	SLIT3	Negative
**3144**	Female	73	LUAD	HPA051630	SLIT3	Negative
**2208**	Female	67	Normal	HPA027541	SPP1	Negative
**2268**	Female	49	Normal	HPA027541	SPP1	Negative
**3076**	Male	20	Normal	HPA027541	SPP1	Low Positive
**1847**	Male	64	LUAD	HPA027541	SPP1	Negative
**2585**	Male	63	LUAD	HPA027541	SPP1	Negative
**3003**	Male	49	LUAD	HPA027541	SPP1	Negative
**3048**	Female	67	LUAD	HPA027541	SPP1	Negative
**3052**	Female	51	LUAD	HPA027541	SPP1	Negative
**3391**	Female	70	LUAD	HPA027541	SPP1	Negative
**1678**	Female	57	Normal	HPA069474	AGER	Positive
**4797**	Male	78	Normal	HPA069474	AGER	Positive
**4840**	Female	43	Normal	HPA069474	AGER	Positive
**1907**	Male	73	LUAD	HPA069474	AGER	Low Positive
**1932**	Female	57	LUAD	HPA069474	AGER	Low Positive
**2003**	Female	61	LUAD	HPA069474	AGER	Low Positive
**3003**	Male	49	LUAD	HPA069474	AGER	Low Positive
**3144**	Female	73	LUAD	HPA069474	AGER	Low Positive
**4923**	Male	57	LUAD	HPA069474	AGER	Low Positive
